# Low seroprevalence of hepatitis C among children at the Princess Marie Louis Children’ s Hospital in Accra, Ghana

**DOI:** 10.11604/pamj.2021.40.158.29524

**Published:** 2021-11-16

**Authors:** Kwabena Obeng Duedu, Donzala Asomah, Seraphine Kugbemanya, Theophilus Korku Adiku

**Affiliations:** 1 Department of Biomedical Sciences, University of Health and Allied Sciences, Ho, Ghana

**Keywords:** Hepatitis C virus, children, liver cirrhosis, cancer

## Abstract

Hepatitis C is a leading cause of chronic hepatitis and causes severe health problems in areas where prevalence is high. Ghana is noted for a relatively high sero-prevalence of hepatitis C virus infection. However, there is very little data on prevalence of hepatitis C virus (HCV) among children in Ghana, and what data is available indicates very low prevalence rate. We conducted a cross-sectional study to determine the sero-prevalence and associated pre-disposing risk factor for HCV infection among children attending the Princes Marie Louis Children´s Hospital in Accra. Two hundred archived blood samples from a previous study were retrieved and tested for the presence of HCV antibodies using a dipstick test kit. Out of the 200 samples tested, one (1) tested positive for HCV antibodies giving a prevalence of 0.5% among the study group. The results show that there is potentially a very low prevalence of hepatitis C among Ghanaian children. Hence, the higher prevalence among adults usually seen is often due to infection later in life. Obtaining an appropriate vaccine early in life could thus help prevent people from getting infected in later life.

## Introduction

The World Health Organization estimates that around 71 million people in the world are infected with chronic hepatitis C infection [1]. Children are particularly at risk and, HCV in children is often asymptomatic, and a screening policy should be considered as a valuable approach for this age group. HCV is transmitted mainly through percutaneous exposure. In children, generally, perinatal (vertical) acquisition of HCV is the most prevalence route of transmission, with about 5% of children born to mothers with HCV viremia having HCV through vertical transmission [2]. Even though, current estimate records a reduction in the burden because of a decreased prevalence rate among children, additional information is required to control the infection.

Approximately 399,000 people die each year from hepatitis C, mostly from cirrhosis and hepatocellular carcinoma (HCC) [3]. Studies have indicated that, though majority of cases of HCV infections acquired in childhood do not look harmful, about 25% of them could develop liver cirrhosis and liver failure in childhood and infrequently HCC after 20-30 years following acute infections [4]. Paediatric HCV infection has generally not been given much attention, particularly in developing countries, where the healthcare costs including economic impact for the HCV-infected children as well as their family contacts are high. Few studies have been reported on the seroprevalence of HCV infections in Ghana. Chronic HCV infection among the greater Accra regional population of Ghana has been reported to be 6.4% [5].

The lack of data on the HCV status in children has disallowed health policy- and decision-makers from having a clear picture of the situation pertaining to HCV infection in children. Hence, the objective of this study was to determine the prevalence of HCV infection and some factors which pre-dispose children to its acquisition in Accra, Ghana. This study thus was to provide very important baseline data to generate critical information needed to formulate effective policies for the prevention of HCV in the Ghanaian population. It was also designed to generate data to provide information for education on preventive measures to be taken to reduce the high prevalence of HCV among the adult population.

## Methods

A hospital-based retrospective study using blood samples and records from a previous study [6] was conducted. Two hundred (200) archived samples were selected randomly and screened for hepatitis C. The samples were tested for the presence of HCV specific antibodies, using an HCV dipstick test kit following the manufacturer´s instructions (Wondfo, Guangzhou, China). Briefly, the dipsticks, patient´s samples and controls were left on the bench to come to room temperature (22-25°C). Each dipstick was labelled with laboratory ID as indicated on the various samples. A drop of thawed whole blood was dispensed onto the tip of the test strip. Two drops of sample diluent were added and incubated for 15 minutes after which the results were read. Data was entered into Microsoft Excel and analysed using IBM SPSS version 25 and GraphPad Prism version 8.

In addition to the ethical clearance for the original study [6], clearance was also obtained from the research ethics committee of the University of Health and Allied Sciences, Ho, Ghana (Ref. No. UHAS-REC A.7 [156] 19-20) prior to the commencement of this study. Informed consent was obtained prior to inclusion in the original study.

## Results

**Figure 1:**
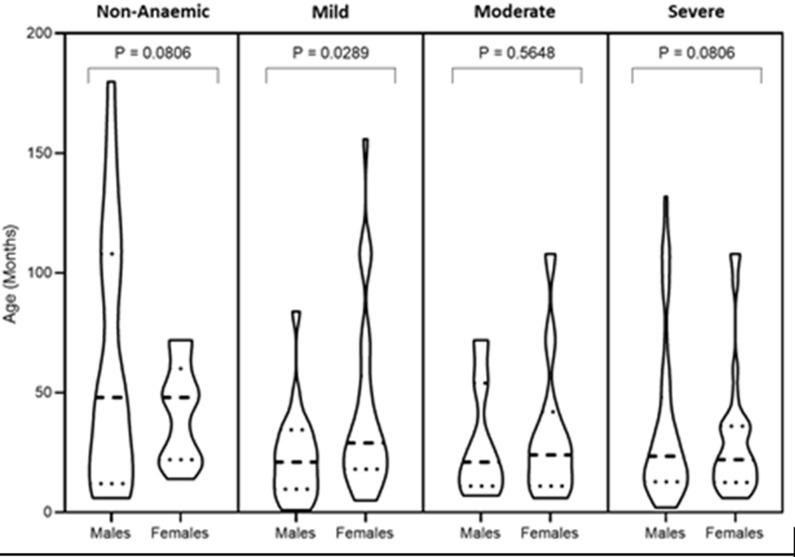
distribution of Hb concentrations among males and females in cohorts of mild, moderate and severe anaemia as well as non-anaemic children

Out of the 200 samples, one tested positive for anti-HCV giving an overall seroprevalence of 0.5%. The single anti-HCV positive sample was from a 19-month-old female with Hb 130 g/L (non-anaemic). The Hb concentration of the participants were classified into non-anaemic, mild, moderate and severe anaemia. The standard deviations of the haemoglobin concentrations among the various cohorts grouped by gender were not equal (non-amaemic SD = 53.05 (male) and 19.83 (female); mild SD = 19.28 (male) and 37.70 (female); moderate SD = 34.06 (male), 28.16 (female); severe SD = 23.60 (male), 30.89 (female)), hence the unpaired t-test with Welch´s correction was used to determine the differences in Hb between gender in the various cohorts. Except for the mild cohort, there was no statistically significant difference (t-test) between the Hb concentration of males and females in the remaining cohorts (Figure 1).

The Kendall´s tau b correlation test was performed to determine whether there was any form of dependence between Hb concentration and ages. There was statistical dependence based on the tau coefficient for Hb concentrations and ages for both males and females (p=0.001 (males/Hb) and p=0.007 (females/Hb)). Overall, in this randomly selected cohort of patients, the majority of anaemic cases were moderate representing 43.6%. The severely anaemic cases constituted the lowest population representing 11.3% whereas 23.5% were with mild anaemia. The datasets used and analysed in the study are available from the corresponding author on reasonable request.

## Discussion

Prevalence of hepatitis C in children in most places is relatively low compared to that of adults. In Nigeria, a HCV seroprevalence of 2% has been reported among children in Ibadan [7]. In the Upper West Region of Ghana, the seroprevalence of HCV in children has been reported to be 2.9% [8]. In one of the largest modelling studies of HCV in children, the global estimate for viraemic prevalence in the paediatric population aged 0-18 years was estimated to be 0.13% with a 95% uncertainty interval of 0.08-0.16. The study also found that HCV prevalence increased with age in all countries and territories with the prevalence in women of childbearing age being the strongest predictor of HCV prevalence in children aged 0 - 4 years [9]. The result from this study thus is not far from the bigger picture. Low prevalence of HCV in children may be due to improved conditions for childbirth and the patronage of hospitals by pregnant mothers.

In the last decade, the World Health Organization has championed various interventions to reduce the practice and patronage of untrained birth attendants in Ghana and other countries. In responding to this, Ghana has put in efforts to train Traditional Birth Attendants (TBAs) who were believed to be using unhygienic means to do deliveries to become Skilled Birth Attendants (SBAs) under the supervision of the Ghana Health Service [10]. It is believed that, use of unsterilized devices for deliveries and circumcision of children can transmit HCV and other infectious diseases [10,11]. Hence, the reduction in the practices of TBA particularly in urban settings like Accra [12] where the study was conducted could be a major reason for the low seroprevalence. Additionally, the Ghana Health Service has made hepatitis B and C testing part of antenatal care. Mothers who are affected when detected are counselled and managed to reduce the potential of transmission to the child [13]. This proactive measure is also another potential reason for the low prevalence. It is therefore important to pursue measures that can contribute to total elimination of the virus.

The importance of HCV infection in children cannot be overemphasised. The national prevalence of HCV in Ghana as reported from a systematic review and meta-analysis of twenty-four studies was found to be 3% [5]. Paediatric HCV has the potential to drive the HCV prevalence in the adult population [14] and hence, it needs the utmost attention. There is a need to institute active surveillance programmes to monitor HCV infection in children. In Ghana and most other developing countries, there are very few to none national programmes in this perspective, hence data largely comes from individual research programmes. This study serves as one of the very few providing an insight into this important public health problem. Although the relatively low seroprevalence is good for public health, it is important to regularly undertake larger multicentre studies to look at the extent of the problem.

## Conclusion

There is a relatively low seroprevalence of HCV in the study population. Whilst efforts are made to develop vaccines and preventive measures to curb HCV globally, it is therefore important to replicate the study in larger populations to determine the current burden to guide public health response against HCV in children.

### What is known about this topic

- About 25% of HCV cases acquired in childhood could develop into liver cirrhosis and liver failure in childhood and infrequently hepatocellular carcinoma after 20-30 years following acute infections;
- The global estimate for viraemic prevalence in the paediatric population aged 0-18 years has been reported to be 0.13% (95% uncertainty interval = 0.08 - 0.16).

### What this study adds

- The seroprevalence of HCV in a subset of Ghanaian children in Accra is 0.5% which is lower than what was previously reported in another cohort of Ghanaian children (2.9%; 2016) and the national seroprevalence estimated from a systematic review and meta-analysis study (3%; 2016);
- HCV infection was not associated with anaemia and the likelihood of any of the children receiving transfusion.
